# Survival of colorectal cancer patients in Brunei Darussalam: comparison between 2002–09 and 2010–17

**DOI:** 10.1186/s12885-021-08224-6

**Published:** 2021-04-30

**Authors:** Shirley H. F. Lee, Hanif Abdul Rahman, Nadiah Abidin, Sok King Ong, Elvynna Leong, Lin Naing

**Affiliations:** 1 PAPRSB Institute of Health Sciences, Universiti Brunei Darussalam, Tungku Link Road, Bandar Seri Begawan, BE1410 Brunei Darussalam; 2 School of Medicine, National University of Ireland Galway, University Road, Galway, H91 TK33 Ireland; 3 Non-Communicable Diseases Prevention Unit, Ministry of Health, Commonwealth Drive, Bandar Seri Begawan, BB3910 Brunei Darussalam; 4 Early Detection & Cancer Prevention Services, Pantai Jerudong Specialist Centre, Bandar Seri Begawan, BG3122 Brunei Darussalam; 5 Faculty of Science, Universiti Brunei Darussalam, Tungku Link Road, Bandar Seri Begawan, BE1410 Brunei Darussalam

**Keywords:** Colorectal cancer, Survival rate, Survival time, Prognosis, Brunei Darussalam, Cancer registry

## Abstract

**Background:**

Colorectal cancer (CRC) is a major cause of cancer-related mortality worldwide. It is the second leading cause of cancer death in men and women in Brunei Darussalam in 2017, posing a major burden on society.

**Methods:**

This retrospective cohort study (*n* = 1035 patients diagnosed with CRC in Brunei Darussalam from 1st January 2002 until 31st December 2017) aims to compare the overall survival rates of CRC patients (2002–2017), to compare survival rates between two study periods (2002–2009 and 2010–2017) and to identify prognostic factors of CRC. Kaplan-Meier estimator and log-rank tests were performed to analyse the overall survival rates of CRC patients. Multiple Cox regression was performed to determine the prognostic factors of CRC with adjusted hazard ratios (Adj. HRs) reported.

**Results:**

The 1-, 3- and 5-year survival rates of CRC patients are 78.6, 62.5, and 56.0% respectively from 2002 to 2017. The 1-, 3-, and 5-year survival rates of CRC patients for 2002–2009 are 82.2, 69.6, and 64.7%; 77.0, 59.1, and 51.3% for 2010–2017 respectively. A significant difference in CRC patients’ survival rate was observed between the two study periods, age groups, ethnic groups, cancer stages, and sites of cancer (*p* < 0.05). The Adjusted Hazard Ratios (Adj. HRs) were significantly higher in the 2010–17 period (Adj. HR = 1.78, *p* < 0.001), older age group ( ≥ 60 years) (Adj. HR = 1.93, *p* = 0.005), distant cancer (Adj. HR = 4.69, *p* < 0.010), tumor at transverse colon and splenic flexure of colon (Adj. HR = 2.44, *p* = 0.009), and lower in the Chinese(Adj. HR = 0.63, *p* = 0.003).

**Conclusion:**

This study highlights the lower survival rates of CRC patients in 2010–2017, Malays, older patients, distant cancer, and tumors located at the latter half of the proximal colon (transverse colon), and predominantly LCRC (splenic flexure, descending colon, sigmoid colon, overlapping lesion colon and colon (NOS), as well as the rectosigmoid junction and rectum (NOS)). Age, ethnicity, cancer stage, and tumor location are significant prognostic factors for CRC. These findings underscore the importance of public health policies and programmes to enhance awareness on CRC from screening to developing strategies for early detection and management, to reduce CRC-associated mortality.

## Introduction

Colorectal cancer (CRC) is the third most commonly diagnosed cancer (10.2%) and the second leading cause of cancer death (9.2%) in men and women worldwide [1]. The high incidence (positively associated with human development index), coupled with the substantial increasing incidence rates of CRC is a major challenge globally [2].

Brunei Darussalam is a high income country [3] in Southeast Asia, with a population of 421,300 in 2017, comprising predominantly Malays (including the indigenous groups: Belait, Bisaya, Brunei, Dusun, Kedayan, Murut and Tutong) (65.8%), Chinese (10.2%) and “Others” (23.9%) [4]. Healthcare is highly accessible in Brunei with services free to all citizens and permanent residents (76.8 and 7.8% of the population respectively). Recent data shows that non-communicable diseases (cancer, heart diseases, diabetes mellitus and cerebrovascular diseases) contributed to 52% of total deaths in Brunei [4], where cancer is the leading cause of death with around 20% of all annual deaths [4–7]. High-income Asia Pacific, including Brunei has one of the highest age-standardised incidence rate (ASR) (per 100 000 person-years) for CRC globally. The ASR of CRC in Brunei has increased by 40.5% (16.6 to 66.7), from 31.2% (26.7 to 36.4) in 1990 to 43.8% (39.8 to 48.6) in 2017, compared to the global average increase of 9.5% (4.5–13.5) (from 21.2% (20.7 to 21.9) to 23.2% (22.7 to 23.7) in 1990 and 2017 respectively) [2], with colon, rectum and anus cancer being the leading cause of cancer death in men (22.5%) and the second leading cause of cancer death in women (14.2%) in Brunei in 2017 [4], highlighting the burden of CRC. The mean age of CRC diagnosis in Brunei Darussalam was 59.3 ± 14.6 years old [8].

The CRCs are a very complex and heterogeneous group of diseases due to the involvement of various mutations and mutagens [9]. The multi-step model of colorectal tumorigenesis states that CRC development often involve the mutational activation of an oncogene combined with the loss of several genes that normally suppress tumorigenesis [10]. Heritability estimates of CRC (from twin and family studies) range from 12 to 35% [11], with around 5% of cases associated with well-characterized familial mutations while the etiologies of the remaining inherited CRCs are not fully known [12]. Although the detailed mechanisms behind CRC development remains incomplete, dietary and lifestyle factors have been implicated [13]. Adaptation of a “Westernized” diet (defined by high intake of refined carbohydrates, added sugars, fats, and animal-source foods [14]), physical inactivity or a sedentary lifestyle, smoking, and comorbidity such as obesity, have been associated a higher CRC risk [15].

Early diagnosis of CRC improves survival, with 5-year survival rate of early stage cancer patients as high as 90% [16–18]. Major interventions to reduce mortality from colorectal cancer include the removal of polyps and early detection interventions with screening tests such as colonoscopy, flexible sigmoidoscopy, faecal occult blood testing (FOBT), and faecal immunochemical testing (FIT) [2]. Previously, colonoscopy and FIT were practiced in Brunei on an opportunistic basis. A national screening programme with screening guidelines for CRC was launched in 2019. Screening is recommended for those between the ages 50–75 years with FIT or/and colonoscopy (FIT every 2 years if negative; colonoscopy for those with positive FIT or for those who opted for scope, scope every 10 years if negative); individuals < 50 years with risk factors for CRC will be offered colonoscopy (every 3–5 years depending on risk factors); individuals aged 76–85 years old are not automatically screened but are offered colonoscopy every 10 years if they are deemed fit for scope and if negative for FIT; if they are unfit for scope, they will undergo FIT every 2 years if FIT negative); screening is not recommended for individuals ≥ 86 years old [19].

Clinical survival data of CRC patients will be useful to the community to narrow any knowledge gaps in screening, diagnosis and intervention. In this study, the overall survival rates of CRC patients at 1-,3- and 5-year in Brunei Darussalam were analysed and compared between two study periods (2002–2009 and 2010–2017). Due to CRC being a leading cause of cancer-related deaths and its increasing incidence, we also aim to identify some of the prognostic factors associated with CRC as study findings may better inform healthcare professionals, and steer health policy makers towards public health initiatives to prevent and reduce mortality from CRC.

## Materials and methods

### Patients

This retrospective population-based cohort study analysed data of 1035 CRC patients from the Brunei Darussalam Cancer Registry (Ministry of Health, Brunei Darussalam) who were diagnosed with CRC from the 1st January 2002 to the 31st December 2017. All patients are residents of Brunei Darussalam.

### Data collection

De-identified data were extracted from the Brunei Darussalam Cancer Registry (BDCR), Ministry of Health. Data extracted include patients’ demographics including their age, gender, ethnicity, residential districts, and clinical details such as date of diagnosis, date of death, staging of cancer, morphology, ICD-10 code and the site of cancer.

Participants were categorised by gender (male and female), age groups (< 40, 40–59, ≥ 60 years), ethnicities (Malay, Chinese, and Others), Surveillance, Epidemiology, and End Results (SEER) stages (localised, regional, or distant), sites of cancer (cecum until hepatic flexure, transverse colon until splenic flexure, descending colon until colon (NOS), and rectosigmoid junction until rectum (NOS)), and living status (alive or deceased). Cancer sites were grouped into 4 categories based on risk profile similarity and to ensure sample sizes are valid for statistical analysis. The first category “cecum until hepatic flexure” includes cecum, appendix, ascending colon, and hepatic flexure of colon; the second category “transverse colon until splenic flexure” includes the transverse colon and the splenic flexure of colon; the third category “descending colon until colon (NOS)” includes the descending colon, sigmoid colon, overlapping lesion colon, and the colon (NOS); the fourth category “rectosigmoid junction until rectum” includes the rectosigmoid junction and the rectum (NOS). The data were further categorised into two study periods (2002–2009 and 2010–2017) before statistical analysis.

### Statistical analysis

Data was imported from a Microsoft Excel sheet and analysed with a statistical software R (Version 4.0.2) and RStudio (Version 3.4.3) for Windows. Descriptive statistics were computed for study variables, followed by univariate Chi-square (χ^2^) test for independence to explore association of variables with CRC between the two study periods (2002–2009 and 2010–2017). Survival rate or overall survival, in this study, is defined by the length of time that patients diagnosed with CRC (from the date of diagnosis of CRC) are still alive. Survival rate analysis of CRC patients at 1, 3, and 5-year interval for different gender, age groups, ethnic groups, stages of cancer, sites of cancer, and study periods were calculated using the Kaplan-Meier method and survival curves were plotted accordingly. Log-rank tests were used to compare the survival rates of CRC patients between gender, age groups, ethnic groups, stages of cancer, sites of cancer, and between two different study periods. Multiple Cox regression was performed to determine the prognostic factors of CRC. Hazard ratios (HRs) and adjusted HRs (Adj. HRs) and their 95% confidence intervals (CIs) were also reported. *p*-value was set at < 0.050 for statistical significance.

## Results

### Characteristics of CRC patients

One thousand thirty-five CRC patients were included in the analysis. There were 303 patients from 2002-2009 and 732 patients fromduring 2010–2017. The demographic and clinical characteristics of CRC patients in Brunei Darussalam are presented in Table 1.

**Table 1:** Demographic and clinical characteristics of colorectal cancer patients (*n* = 1035)

Characteristics	Overall		2002–2009		2010–2017		***p*** value^**a**^
	***n***	(%)	***n***	(%)	***n***	(%)	
**Age**							
**< 40 years**	82	(7.9)	21	(6.9)	61	(8.3)	0.389
**40–59 years**	436	(42.1)	121	(39.9)	315	(43.0)	
**≥ 60 years**	517	(50.0)	161	(53.1)	356	(48.6)	
**Gender**							
**Male**	586	(56.6)	172	(56.8)	414	(56.6)	1.000
**Female**	449	(43.4)	131	(43.2)	318	(43.4)	
**Ethnicity**							
**Malay**	751	(72.6)	213	(70.3)	538	(73.5)	0.172
**Chinese**	228	(22.0)	77	(25.4)	151	(20.6)	
**Others**	56	(5.4)	13	(4.3)	43	(5.9)	
**District**							
**Brunei-Muara**	668	(67.5)	209	(71.6)	459	(65.8)	0.143
**Tutong**	123	(12.4)	29	(9.9)	94	(13.5)	
**Belait**	176	(17.8)	45	(15.4)	131	(18.8)	
**Temburong**	23	(2.3)	9	(3.1)	14	(2.0)	
**SEER stage**							
**Localised**	264	(33.9)	91	(39.7)	173	(31.5)	**0.001**
**Regional**	242	(31.1)	50	(21.8)	192	(35.0)	
**Distant**	272	(35.0)	88	(38.4)	184	(33.5)	
**Sites of cancer**							
**Cecum**	11	(1.1)	1	(0.3)	10	(1.4)	0.131
**Appendix**	20	(1.9)	4	(1.3)	16	(2.3)	
**Ascending colon**	39	(3.8)	16	(5.2)	23	(3.1)	
**Hepatic flexure of colon**	14	(1.4)	3	(1.0)	11	(1.5)	
**Transverse colon**	28	(2.7)	10	(3.3)	18	(2.4)	
**Splenic flexure of colon**	9	(0.9)	1	(0.3)	8	(1.1)	
**Descending colon**	38	(3.7)	7	(2.3)	31	(4.3)	
**Sigmoid colon**	186	(18.0)	55	(18.0)	131	(17.7)	
**Overlapping lesion colon**	16	(1.5)	3	(1.0)	13	(1.8)	
**Colon, NOS**^b^	224	(21.6)	78	(25.9)	146	(20.1)	
**Rectosigmoid junction**	123	(11.9)	29	(9.5)	94	(12.8)	
**Rectum, NOS**^b^	327	(31.6)	96	(31.8)	231	(31.5)	

^a^ Chi-square (χ^2^) test (comparing two periods)

^b^*NOS* Not otherwise specified

Half of the patients (50.0%) were 60 years old and/or above, followed by patients aged 40 to 59 years old (42.1%). The youngest patients were 19 years old for the 2002–2009 period and 11 years old for the 2010–2017 period (data not shown). More than half (56.6%) of the patients were males. Over 70% were Malays while 22.0 and 5.4% were of Chinese ethnicity and other ethnicities respectively. Majority of patients reside in Brunei-Muara district (67.5%), followed by Tutong (12.4%), Belait (17.8%), and Temburong (2.3%). Thirty-three point 9 % (33.9%), 31.1 and 35.0% of patients were diagnosed at localised, regional and distant cancer stages respectively. Fifty-six point 5 % (56.5%) of the patients had primary tumor sites within the colon (cecum (1.1%), appendix (1.9%), ascending colon (3.8%), hepatic flexure of colon (1.4%), transverse colon (2.7%), splenic flexure of colon (0.9%), descending colon (3.7%), sigmoid colon (18.0%), overlapping lesion colon (1.5%) and colon (NOS) (21.6%)), while tumors in the rectum (rectosigmoid junction (11.9%) and rectum (NOS) (31.6%)) account for 43.5% of total CRC cases. By the end of the study period, 49% of the patients were still alive (data not shown). Upon comparing the two study periods (2002–2009 and 2010–2017), only SEER stage was significantly different (*p* = 0.001). In 2010–2017, there were less distant cases (33.5% versus 38.2%) and localised cases (31.5% versus 39.7%) but more regional cases (31.5% versus 21.8%) compared to 2002–2009.

### Overall survival rates of CRC patients

**Fig. 1:**
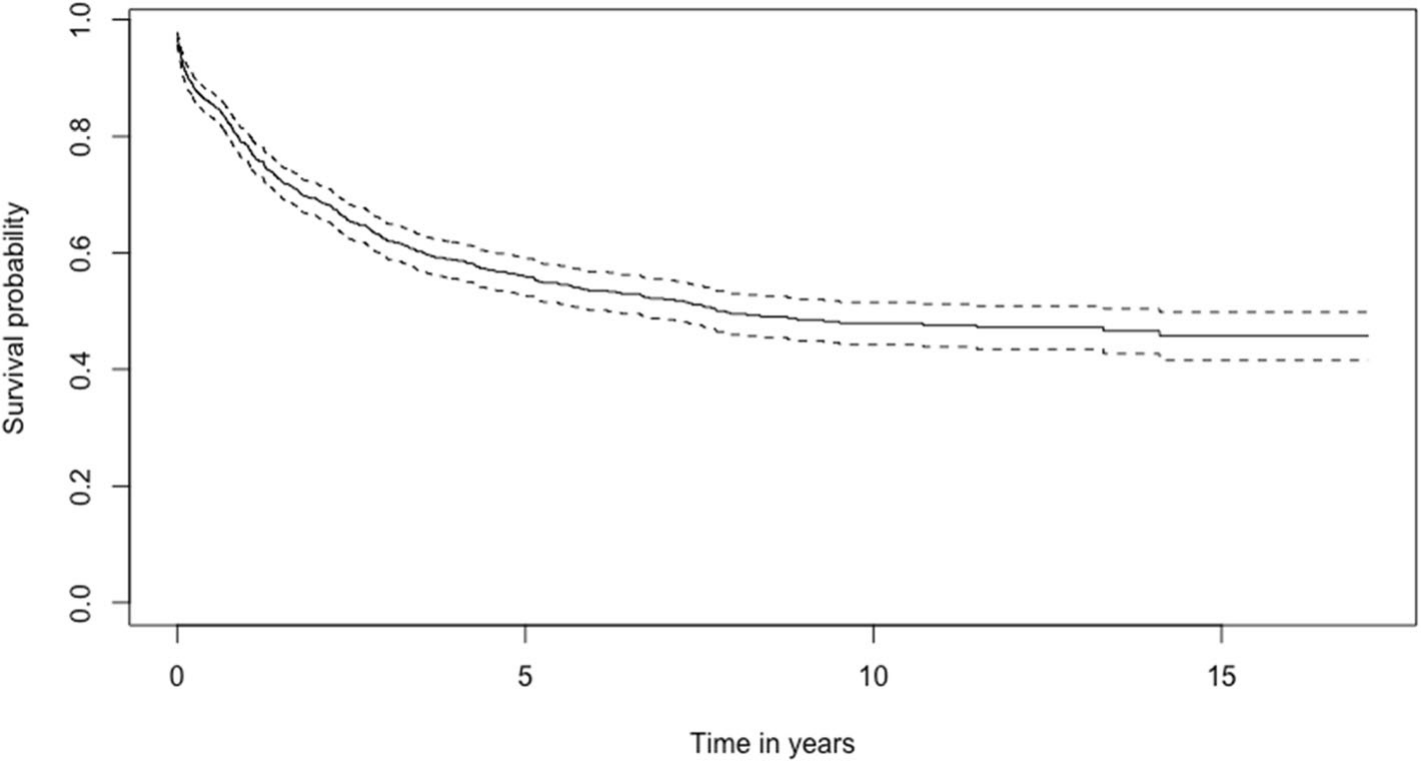
Kaplan-Meier Survival Curve of Colorectal Cancer Patients (2002–2017)

**Fig. 2:**
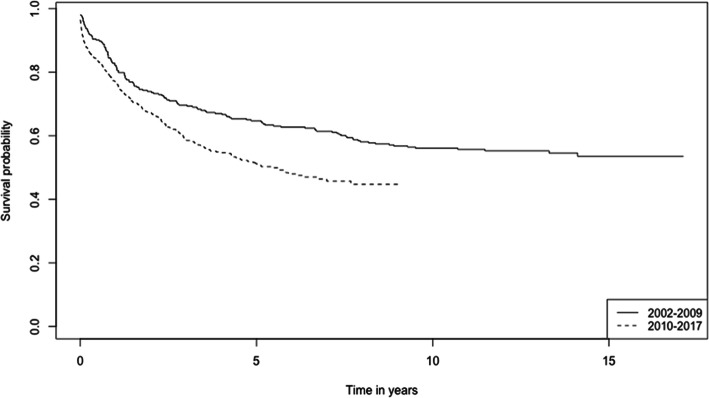
Kaplan-Meier Survival Curves of Colorectal Cancer Patients (comparing two study periods)

**Fig. 3:**
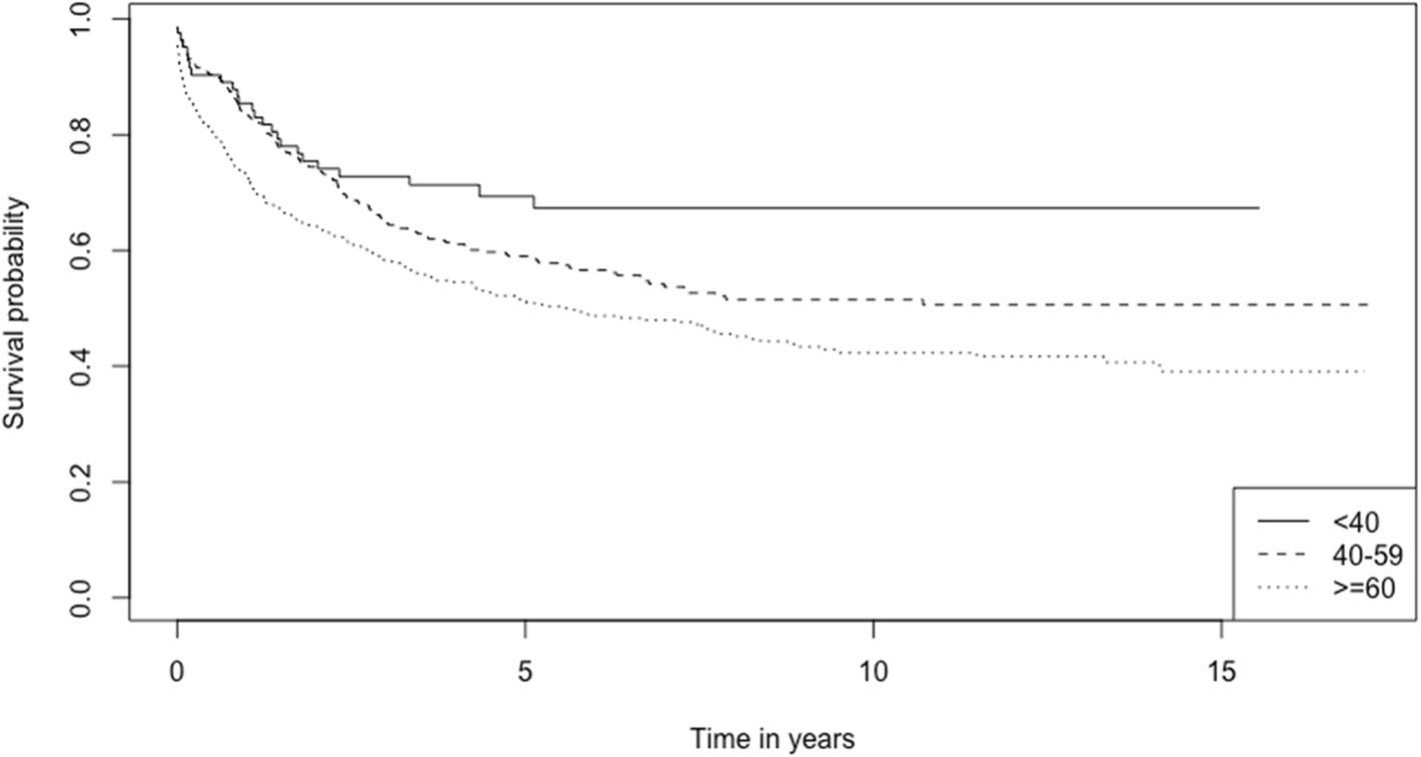
Kaplan-Meier Survival Curves of Colorectal Cancer Patients by Age Groups

**Fig. 4:**
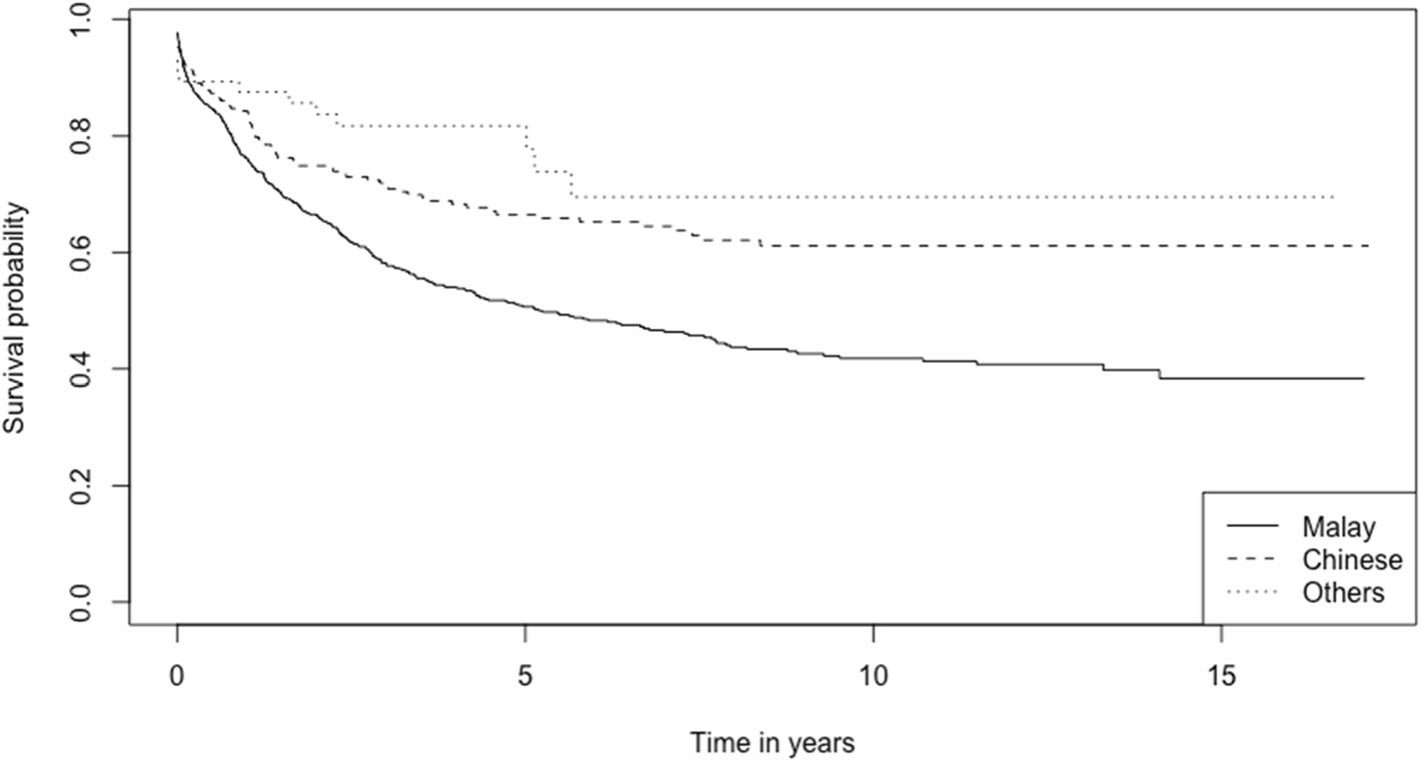
Kaplan-Meier Survival Curves of Colorectal Cancer Patients by Ethnic Groups

**Fig. 5:**
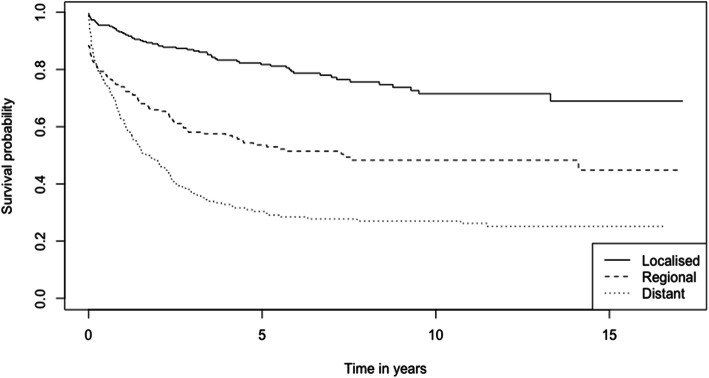
Kaplan-Meier Survival Curves of Colorectal Cancer Patients by SEER Staging

**Fig. 6:**
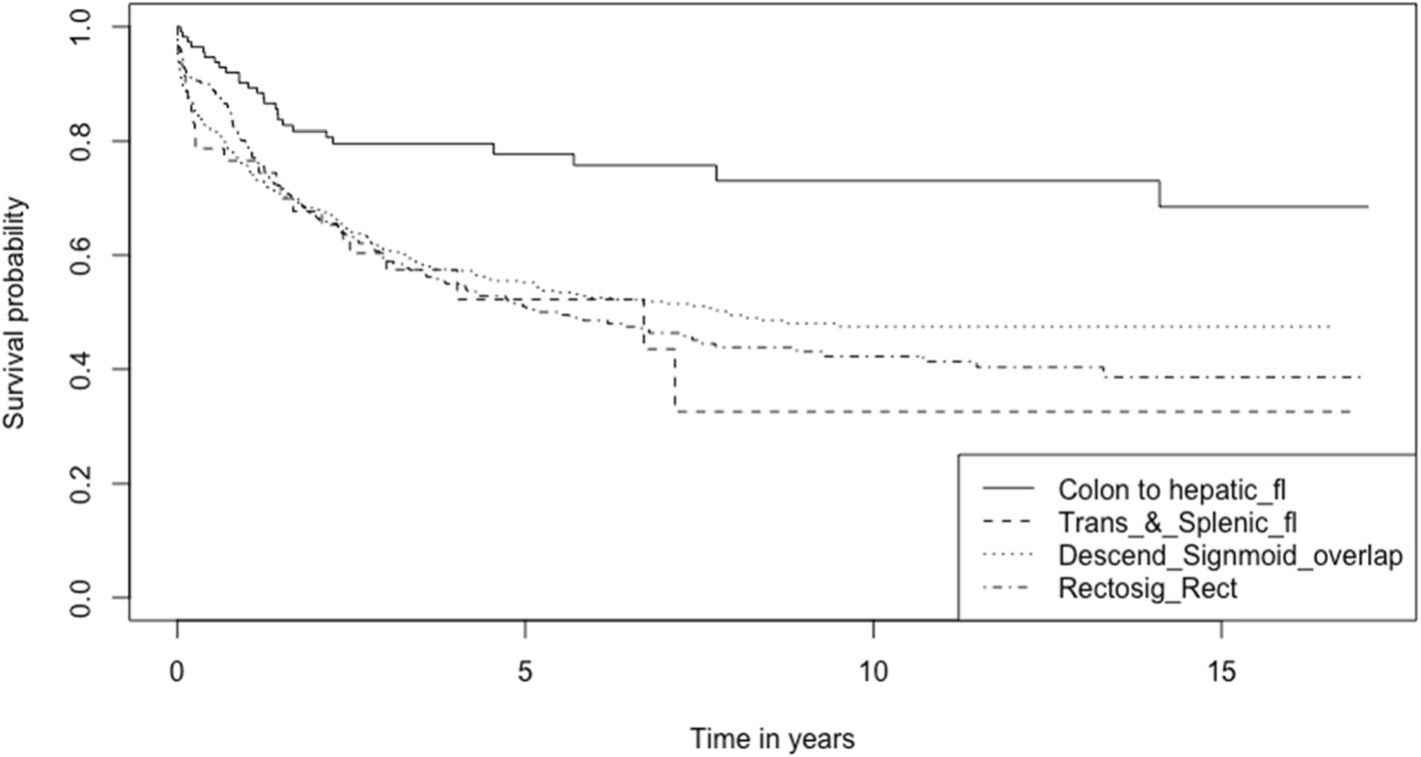
Kaplan-Meier Survival Curves of Colorectal Cancer Patients by Sites of Cancer

The 1-, 3-, and 5-year survival rates of CRC patients from the period 2002–2017 are 78.6, 62.5, and 56.0% respectively (Table 2). The overall Kaplan-Meier survival curve is presented in Fig. 1. Comparing the two study periods, patients diagnosed in 2002–2009 have higher 1-, 3-, and 5-year survival rates (82.2, 69.6, and 64.7% respectively) than patients diagnosed between 2010 and 2017 (77.0, 59.1, and 51.3% respectively) (*p* < 0.001) (Table 2). Kaplan-Meier survival curves from the two study periods are shown in Fig. 2. Log-rank test showed that age (*p* < 0.001), ethnicity (*p* < 0.001), cancer (SEER) stages (*p* < 0.001) and sites of cancer (*p* < 0.001) significantly affect the survival of CRC patients (Table 2). Survival curves of CRC patients by age groups, ethnicity, cancer stages, and sites of cancer are presented in Figs. 3, 4, 5, and 6 respectively.

**Table 2:** Comparison of survival by periods and other characteristics (*n* = 1035)

Characteristic	1-year survival	3-year survival	5-year survival	***χ***^***2***^ (***df***)	***p*** value^**a**^
**Overall**	78.6%	62.5%	56.0%	–	–
**Two periods**					
**2002–09**	82.2%	69.6%	64.7%	13.9 (1)	< 0.001
**2010–17**	77.0%	59.1%	51.3%		
**Age in year**					
**< 40**	85.4%	72.8%	69.4%	16.3 (2)	< 0.001
**40–59**	83.5%	65.3%	59.0%		
≥ **60**	73.3%	58.3%	51.2%		
**Gender**					
**Male**	78.8%	62.4%	55.7%	0 (1)	1.000
**Female**	78.2%	62.5%	56.2%		
**Ethnicity**					
**Malay**	76.2%	58.2%	50.7%	27.1 (2)	< 0.001
**Chinese**	84.2%	71.4%	66.5%		
**Others**	87.5%	81.7%	81.7%		
**District**					
**Brunei-Muara**	80.2%	65.6%	58.7%	0.4 (3)	0.900
**Tutong**	82.1%	60.9%	52.3%		
**Belait**	81.8%	62.2%	56.7%		
**Temburong**	73.9%	56.2%	56.2%		
**SEER stage**					
**Localised**	92.8%	86.9%	81.7%	130 (2)	< 0.001
**Regional**	74.0%	58.1%	53.6%		
**Distant**	62.5%	37.1%	30.4%		
**Sites of cancer**					
**Cecum_Hepatic**^b^	90.2%	79.6%	77.8%	19.3 (3)	< 0.001
**Transverse_Sple**^c^	76.6%	60.3%	52.2%		
**Descend_Col**^d^	75.8%	61.0%	55.2%		
**Rectosig_Rect**^e^	79.5%	59.6%	51.0%		

^a^Log-rank test; *χ*^*2*^ Chi-square statistic, *df* degree of freedom

^b^Includes cecum, appendix, ascending colon and hepatic flexure of colon

^c^Includes transverse colon and splenic flexure of colon

^d^Includes descending colon, sigmoid colon, overlapping lesion colon and colon (not otherwise specified (NOS))

^e^Includes rectosigmoid junction and rectum (NOS)

### Prognostic factors

Multiple Cox regression analysis shows that age groups, ethnicity, cancer (SEER) stages, and sites of cancer are significant prognostic factors for CRC (*p* < 0.050) (Table 3). Patients diagnosed with CRC in 2010–2017 had a significantly poorer prognosis [Adj. HR = 1.78, 95% CI: 1.38, 2.29, *p* < 0.001] than those diagnosed in 2002–2009. The oldest age group (≥ 60 years) has a significantly poorer prognosis [Adj. HR = 1.93, 95% CI: 1.21, 3.06, *p* = 0.005] compared to those below 40 years old, whereas patients aged 40–59 years have comparable prognosis [Adj. HR = 1.46, 95% CI: 0.92, 32.33, *p* = 0.113] relative to patients below 40 years old. Among ethnic groups, the Chinese had significantly better prognosis [Adj. HR = 0.63, 95% CI: 0.48, 0.86, *p* = 0.003] than the Malays. Patients with distant cancer have significantly poorer prognosis [Adj. HR = 4.69, 95 CI%: 3.46, 6.36, *p* < 0.001] compared to patients with localized cancer. This trend is also observed in patients with regional cancer [Adj. HR = 2.34, 95 CI%: 1.69, 3.25, *p* < 0.001]. In addition, tumor location significantly affects survival of CRC patients: Patients with tumors in the transverse colon and splenic flexure of colon exhibit the poorest prognosis [Adj. HR = 2.44, 95% CI: 1.25, 4.76, *p* = 0.009] compared to patients with tumors in the cecum until hepatic flexure (includes the cecum, appendix, ascending colon, and hepatic flexure of colon). Patients with tumors in the descending colon to colon (NOS) (descending colon, sigmoid colon, overlapping lesion colon, and colon (NOS)) have the second poorest prognosis [Adj. HR = 2.01, 95% CI: 1.26, 3.20, *p* = 0.003], compared to patients with tumors in the cecum until hepatic flexure. The prognosis of patients with tumors in the rectosigmoid junction and rectum (NOS) [Adj. HR = 2.00, 95% CI: 1.24, 3.24, *p* = 0.005] is comparable to those with tumors in the descending colon until colon (NOS) (relative to patients with tumors in the cecum until hepatic flexure).

**Table 3:** Comparing survival between two periods controlling for other variables using multiple Cox regression

Characteristic	HR	(95% CI)	***p*** value^**a**^	Adj. HR^**b**^	(95% CI)	***p*** value^**a**^
**Two periods**						
**2002–09**	1.00			1.00		
**2010–17**	1.49	(1.21, 1.83)	< 0.001	1.78	(1.38, 2.29)	< 0.001
**Age in year**						
**< 40**	1.00			1.00		
**40–59**	1.42	(0.94, 2.17)	0.094	1.46	(0.92, 2.33)	0.113
**≥ 60**	1.92	(1.27, 2.90)	0.002	1.93	(1.21, 3.06)	0.005
**Gender**						
**Male**	1.00					
**Female**	1.01	(0.84, 1.21)	0.953			
**Ethnicity**						
**Malay**	1.00			1.00		
**Chinese**	0.60	(0.47, 0.76)	< 0.001	0.63	(0.48, 0.86)	0.003
**Others**	0.40	(0.23, 0.70)	0.001	0.30	(0.14, 0.62)	0.001
**District**						
**Brunei-Muara**	1.00					
**Tutong**	1.09	(0.82, 1.46)	0.553			
**Belait**	0.99	(0.77, 1.28)	0.949			
**Temburong**	1.06	(0.57, 2.00)	0.847			
**SEER stage**						
**Localised**	1.00			1.00		
**Regional**	2.80	(2.04, 3.83)	< 0.001	2.34	(1.69, 3.25)	< 0.001
**Distant**	4.86	(3.62, 6.54)	< 0.001	4.69	(3.46, 6.36)	< 0.001
**Sites of cancer**						
**Cecum_Hepatic**^c^	1.00			1.00		
**Transverse_Sple**^d^	2.52	(1.43, 4.45)	0.001	2.44	(1.25, 4.76)	0.009
**Descend_Colo**^e^	2.23	(1.49, 3.35)	< 0.001	2.01	(1.26, 3.20)	0.003
**Rectosig_Rect**^f^	2.42	(1.60, 3.67)	< 0.001	2.00	(1.24, 3.24)	0.005

^a^Wald test; ^b^*HR* Hazard ratio (adjusted (Adj.))

^c^Includes cecum, appendix, ascending colon and hepatic flexure of colon

^d^Includes transverse colon and splenic flexure of colon

^e^Includes descending colon, sigmoid colon, overlapping lesion colon and colon (not otherwise specified (NOS))

^f^Includes rectosigmoid junction and rectum (NOS)

## Discussion

There is a huge incidence gap of CRC across countries and world regions whereby incidence is associated with the level of socioeconomic development. Regions higher on the Human Development Index (HDI) have higher incidence, and regions that are lower on the HDI have a lower incidence [13]. The higher incidence of CRC in Brunei (a high HDI region) [20] may be a reflection of the quality of our healthcare infrastructure and surveillance system. There is a disproportionate (2.42-fold or 141.5%) increase in the number of CRC patients diagnosed in 2010–2019 (732 individuals) compared to 2002–2009 (303 individuals) (the population has increased by only 1.08 fold or 8.6% [6, 21, 22] between the two periods). The introduction of Brunei Darussalam Healthcare Information and Management System (Bru-HIMS), a national and centralised electronic medical health database in late 2012 may have improved medical surveillance and better capture all CRC patients, partially accounting for the spike in numbers in the latter period. The substantial rise in CRC cases from 2002 to 2009 to 2010–2017 may also be due to other factors. Dietary patterns have been strongly implicated in CRC development. Diet can modulate the gut microbiota and their metabolites, epigenetics, inflammation and immune function, and trigger metabolic or hormonal disruption to influence cancer risk [23]. With modernization, the global nutrition transition has rapidly shifted dietary behaviours to heavy reliance on processed foods, increased away-from-home food intake and excessive consumption of edible oils and sugar-sweetened beverages, leading to adverse outcomes [14]. A recent systematic review and meta-analysis reported an association between a Westernized diet and higher risk of CRC [24], with plant-based diets conferring protective effects against CRC [25, 26]. It is noted that a high proportion (91.7%) of the Bruneian adult population do not meet the required consumption of fruits and vegetables [27]. Tobacco smoking has been shown to cause CRC, especially among long-term smokers through formation of polyps in the intestine [28–30]. Although the percentage of smokers in this population remains unknown, a study has reported that 20% of adults in Brunei smoke [27]. Physical inactivity may increase the risk of CRC [31], although the reason behind it is still unclear [32]. A previous study has shown that physical inactivity is prevalent among elderly Bruneians. Another risk factor is obesity, which may be related to physical inactivity and dietary behaviors. Brunei has the highest prevalence of obesity (28.2%) compared to other Southeast Asian countries [27].

Comparing the two periods, there is an increasing proportion of younger CRC patients (< 40 years old, and 40–59 years old) from 46.8 to 51.3%; specifically, the percentage increase of patients < 40 years old is 20.3% from 2002 to 2009 to 2010–2017, and the percentage increase of patients 40–59 years old patients is 7.8% from 2002 to 2009 to 2010–2017). Meanwhile, the proportion of elderly CRC patients ≥ 60 years old has decreased from 53.1% in 2002–2009 to 48.6% in 2010–2017, a percentage decline of 8.5% (for comparison, the percentage proportion of ageing population in Brunei in the latter period has expanded by 72.5% where the proportion of ≥ 55 year olds increased from 6.2% [21, 22] (2002–2009) to 10.7% (2010–2017)) [6]. The rise in young CRC patients (< 40 years old) (20.3% increase in proportion of CRC cases; from 6.9% in 2002–2009 to 8.3% in 2010–2017) calls for awareness on the existence of CRC in younger individuals. Some studies have recommended a lower screening age, with non-invasive techniques such as the FOBT for high-risk groups < 50 years [33]. With the advent of precision medicine, personalized screening approaches such as genetic profiling of high-risk patients to reveal specific molecular alterations to determine genetic susceptibility and to guide targeted therapy [34, 35], may also benefit survival outcomes. A novel, US FDA (United States Food and Drug Administration)-approved non-invasive stool-DNA test, Cologuard (also known as a multitargeted stool DNA test [MT-sDNA] or FIT-DNA) which tests for both abnormal cancer-associated DNA changes (from CRC or polyp cells that often enters the stool) and blood in the stool without requiring any dietary or drug restrictions may also be considered, as it comes with the flexibility of self-sampling from home [36] for patients’ convenience and preserves privacy.

Advanced stage CRC (Stage 3 or 4) was observed in 68.5% (regional: 35.0%, distal: 33.5%) of our patients in 2010–2017, up from 60.2% in 2002–2009. This may be attributed to several factors such as low awareness of early signs and symptoms of CRC (54.1% of the population are unaware of the signs and symptoms of CRC) [37], fears and denial in the context of Brunei (the free healthcare coverage for citizens and permanent residents (84.6% of the population) in Brunei negates the effects of healthcare expenses as a barrier to seeking diagnosis/treatment). Besides fear and denial of cancer diagnosis, fear of poor prognosis, or fear of embarrassing and unpleasant medical investigations, have also been linked to increase delay in seeking medical advice or treatment. These fears and denial behaviours in turn, are attributed to a lack of knowledge about symptoms, the importance and implications of CRC diagnosis at an early stage, and the availability of diagnostic tools [38]. In addition, those experiencing non-specific CRC symptoms such as weight loss and fatigue tend to be normalized and be disregarded by patients, which may prevent them from seeking timely medical attention. Although advanced stage (regional and distant stages) CRC diagnosis increased in the latter period generally, distant stage CRC cases were relatively lower (33.5% versus 38.4%) whereas regional stage CRC cases were relatively higher (35.0% versus 21.8%) in 2010–2017 compared to 2002–2009. The shift of diagnostic trend from distant stage to regional stage may reflect the improved surveillance in the latter period, post-Bru-HIMS. However, contrary to our expectations (that the improved surveillance would improve early/localised stage diagnosis), there were relatively less CRC patients diagnosed at localised stage (31.5%) in 2010–2017 compared to 2002–2009 (39.7%). The relatively higher proportion of localised stage CRC cancers in the earlier period (2002–2009) may be due to an underestimation of advanced (regional and distant) CRC cases (60.2%), pre-Bru-HIMS. Underestimation of advanced-stage CRC patients in the earlier period (2002–2009) may arise as CRC patients with late-stage diagnosis are more likely to exhibit strong fears and denials and experience unsuccessful or refuse referrals, therefore remain uncaptured before the establishment of the digital healthcare system in 2012. Findings from the Health Screening Program conducted in Brunei Darussalam in 2009 involving government civil servants (FIT-positive) shows poor screening uptake rate, with just over half of the patients agreeing to colonoscopy, suggesting the importance of public measures to fight ignorance and increase awareness. The ‘private nature’ of CRC symptoms also suggests that it is crucial to reduce stigma on conversations around bowel movements with others including healthcare professionals [39]. It will also be useful to characterize patients’ experience, from symptoms’ (bleeding, obstruction, and abdominal/rectal pain [40]) onset to diagnosis, to identify potential barriers and promptly guide future interventions [41].

No significant difference in survival rates between genders were observed in this study, although female CRC patients are significantly younger (< 40 years) than male CRC patients (*p* = 0.002) (data not shown), consistent with a previous 29-year national epidemiological study [42]. However, males account for more than half of the total CRC cases (56.8%) in 2002–2009 and (56.6%) in 2010–2017, concurrent with findings from previous reports of higher incidence of CRCs among males [43, 44]. A recent systematic analysis of global CRC trends from 1990 to 2017 across 195 countries and territories reported high numbers of incidence and deaths among males than females up to the ages of 80–84 years, with the highest rates observed in the oldest age group (≥ 95 years) for both sexes [2]. It has been reported that female CRC patients have a survival advantage compared to male CRC patients, particularly in young and middle-aged patients and patients with localized disease [45]. Factors such as differences in diet and lifestyle [15], and the protective effect of estrogens in females against CRC [46, 47], may explain the male predominance in CRC. Meanwhile, other studies observed no differences in 5-year survival between the sexes [reviewed in [43]]. The association between gender and CRC survival therefore remains to be validated as study findings remain inconsistent [48], due to variations in study design/analysis and differences in CRC subgroupings. Standardizing and harmonizing study designs with large, multi-center, population-based data may be the way forward.

The global heterogeneity in survival rates of CRC patients may be due to the varied lifestyles, environmental, and genetic factors. The 1, 3- and 5-year survival rates of CRC patients from 2002 to 2017 in this study are 78.6, 62.5, and 56.0% respectively. The overall 5-year survival rate for CRC patients in this study (56.0%) is slightly below the average (60%) for Asia [49], with South Korea observing the highest levels of 5-year survival (75%) of CRC patients from 2011 to 2015 [50]. A US-based study of CRC patients diagnosed between 2008 and 2014 has reported average 5-year relative survival rates at 64.5% (across all races investigated), ranging from 57.8 to 66% [9]. Meanwhile, the 5-year net survival for colon cancer across Europe from 2010 to 2014 averages to 60%, ranging from 65% or more in Belgium, Finland, Sweden and Germany to less than 55% in many Central and Eastern European countries including Latvia, Croatia, the Slovak Republic, Romania, Bulgaria and Poland. These countries also have lower 5-year net survival for rectal cancer [51]. The lower overall 5-year survival rate for CRC patients in our study may be due to the high proportion of advanced stage CRC (particularly distant stage diagnosis) at 35.0% from 2002 to 2017 compared to 15.6% (2006–2015 data) in South Korea [50], 21.2 to 39% (1995–2011 data) between different regions in Malaysia and 23.2% in Singapore (2007–2011 data) [52], suggesting the importance of early detection on improved survival.

Our study also demonstrates that the 5-year survival rate of CRC patients is significantly lower in 2010–2017 (51.3%) compared to 2002–2009 (64.7%). This may be attributed to a larger number of unrecorded and/or undiagnosed CRC mortalities in 2002–2009 (pre-Bru-HIMs), leading to an overestimation of surviving patients in the earlier period. In addition, Bru-HIMS-facilitated alterations in the coding mechanism for causes of deaths may also contribute to improved/accurate case registration in the latter period (2010–2017). Epidemiological studies have shown an increased risk of CRC in patients with inflammatory bowel disease (IBD) [53]. The incidence of IBD in Brunei has increased from 0.28 per 100,000 population in 2004 to 3.08 per 100,000 population in 2016 [54] . However, the proportion of patients with IBD who transitions to CRC remains to be determined. A poorer prognosis in the latter period may also be attributed to treatment refusal or high dropout rates among patients. Advanced-stage cancer, feeling discouraged or depressed from the cancer worsening, and pre-existing catastrophic illnesses make patients more likely to refuse treatment [55], which may contribute to a lower 5-year survival rate.

This study shows that CRC patients < 40 years old have a higher 5-year survival rate than those aged ≥ 60 years old (69.4% versus 51.2% respectively). Adj. HRs indicate that patients ≥ 60 years old have the highest risk of mortality, followed by the 40 to 59 years old group. This is contrary to a previous report, which shows that age does not impact survival rates of CRC patients significantly [48]. The average life expectancy (males and females) in Brunei has increased from 76.6 years between 2002 and 2009 [56] to 77.6 years between 2010 and 2017 [19], suggesting that the increased mortality risk among the elderly group is not attributed to a general reduction in life expectancy. The increasing proportion of ageing population nationally [6, 21, 22] and globally [57] calls for concern to address ways to reduce mortality among the elderly CRC patients. The presence of more advanced comorbidities in elderly CRC patients [58, 59], may also explain their lower survival rate. Malnutrition, a frequent physical manifestation of gastrointestinal cancers which is particularly common in older adults, have potential negative repercussions on quality of life, functional status, treatment tolerance, and prognosis [60]. However, nutrition post-cancer diagnosis especially in elderly patients remains understudied [61] and knowledge gaps remain to be closed.

Malaysian and Bruneian studies have reported a higher incidence of CRC among the Chinese [48, 62, 63]. As mentioned earlier, the burden of colorectal cancer has been attributed to dietary risks, and lifestyle factors including alcohol consumption, and smoking [2]. Cultural and lifestyle differences among different ethnicities may contribute to the etiology of CRC. Previous studies show strong evidence of an association between alcohol consumption and colorectal cancer risk [64–66]. It remains to be determined whether alcohol consumption is associated with the increasing incidence of CRC among Bruneian-Chinese (alcohol consumption is forbidden amongst Muslims (predominantly Malays), by the Islamic law in this country). The unequal CRC risk between different ethnic groups within the same region may also be explained by genetic factors that may alter the effects of the environment on disease predisposition [67]. Despite the higher incidence of CRC among the Chinese population in Brunei, they have significantly higher 1-year, 3-year and 5-year survival rates, and a significantly lower risk of death after adjusting for variables, compared to the Malays. In addition, Bruneian-Chinese tend to develop CRC at a slightly later age (60.4 ± 12.7 years) compared to the Malays (59.9 ± 15.2 years) [8]. The disparity in survival rates between CRC patients of differing ethnicities in Brunei are in agreement with findings in Singaporean studies [68, 69] but differs from the findings in Malaysia [48]. It is important to consider that the number of Bruneian-Chinese patients may be underestimated in our study, as they may seek diagnosis and/or treatment overseas [62], thus may not be enrolled in the cancer registry. The reluctance to seek aggressive therapies among the Malay CRC patients may contribute to their low survival rates [68, 69]. Local clinicians also report the tendency of patients to opt for mostly traditional medicine before considering, and therefore delaying conventional treatment (personal communication). It is integral that healthcare professionals in Brunei work to increase targeted awareness in the community regarding CRC symptoms and the importance of early screening and early intervention.

This study shows that distant CRC is associated with the highest risk of death, followed by regional cancer, and localised cancer, consistent with previous reports [9, 31]. Surgical removal of advanced staged tumors with metastatic lesions is challenging [70], therefore, minimizing the number of CRC patients who present with advanced stage diagnosis needs to be emphasised. It is also crucial for physicians and the relevant multidisciplinary team to construct an effective treatment and clinical management plan with supportive care for patients to improve their quality of life.

This study shows that tumor location impacts survival outcome of CRC patients. The definition of right-sided colorectal cancer (RCRC) and left-sided colorectal cancer (LCRC) varies across different studies with respect to tumors in the transverse colon (some studies consider the cecum, ascending colon, hepatic flexure and transverse colon as RCRC; LCRC includes the splenic flexure, descending colon, sigmoid colon and rectosigmoid cancers [71–73]) whereas other studies only include the proximal two-thirds of the transverse colon (alongside cecum, ascending colon and hepatic flexure) as RCRC, with the distal third of the transverse colon (alongside splenic flexure, descending colon, sigmoid colon and rectosigmoid cancers) categorized as LCRC [74, 75]. The effect of CRC tumor subsite on CRC patients’ survival rate of our study differs from previous reports of improved prognosis in LCRC [76–79]. Our study shows that majority of the patients (89.2%) present with LCRC (tumor sites from splenic flexure to the rectum), which tends to associate with lowered 5-year survival rates and higher risk of death. CRC patients with tumors in the first part of the proximal/right-sided colon (cecum until hepatic flexure) have a significantly higher 5-year CRC survival rate (77.8%), compared to patients with tumors in the transverse colon and splenic flexure (5-year survival: 52.2%), patients with tumors in the descending colon, sigmoid colon, overlapping lesion colon and colon (NOS) (5-year survival: 55.2%) and patients with tumors in the rectosigmoid junction until rectum (5-year survival: 51.0%). Patients with tumors in the transverse colon and splenic flexure [Adj. HR = 2.44 (1.25, 4.76); *p* = 0.009], descending colon, sigmoid colon, overlapping lesion colon and colon (NOS) [Adj. HR = 2.01 (1.26, 3.30); *p* = 0.003]) followed by those with tumors in the rectosigmoid junction until rectum (NOS) [Adj. HR = 2.00 (1.24, 3.24); *p* = 0.005]) have significantly higher risk of mortality compared to patients with tumors located from the caecum to the hepatic flexure of colon. RCRC and LCRC have differing embryological origins (right-sided colon arises from the embryologic midgut whereas left-sided colon is derived from the embryologic hindgut) [80], molecular etiologies [81] and significant epidemiological, clinical and histological differences [82, 83]. Microsatellite instability (MSI) is more common in RCRCs. Although tumors showing MSI have an improved prognosis, the good prognosis may be counteracted by the fact that they tend to present at a later stage. In contrast, LCRCs tend to involve p53 mutations, and overexpression of vascular endothelial growth factor which are associated with an adverse prognosis and poor response to fluorouracil-based chemotherapy [82]. RCRC has been associated with poorer survival outcomes as RCRC patients tend to be older [74, 83, 84], and typically present with more advanced, poorly differentiated tumor, with flat morphology, thus more challenging to detect, compared to LCRC patients [76, 80, 81]. Although CRC patients with tumors in the cecum until the hepatic flexure (RCRC) have the highest survival rate compared to CRC patients with other cancer sites, the sample size is significantly smaller (8.2% of total CRC patients) than that of other cancer sites. Therefore, these findings remain to be validated with a larger sample size. The small number of RCRC patients (10.8% of total CRC patients) in this study may also be due to reporting bias, due to challenges in detection as mentioned earlier. As CRC presents differently depending on tumor location and sidedness, screening and awareness efforts should emphasize the different presentations [85]. Screening modality choice may also be affected by the anatomic location of CRC presentation; for instance FOBT is more sensitive for detecting left-sided lesions compared with right-sided lesions [86]. Overall, the survival rate of CRC patients with LCRC and RCRC remain conflicting, and there is insufficient evidence to support the use of tumor location in making decisions about therapy. Therefore, a deeper understanding of the interaction between tumor sub-site and molecular profile may facilitate personalised therapy to improve management of CRC patients [87].

A strength of our study is the use of a population-based national cancer registry where data is centralized and cross-verified from the pathology laboratory, clinical reports, electronic medical records and death registry. However, like any data registry, there are several limitations such as coding inaccuracies due to heavy manual data-recording especially in the early period (2002 to 2009) prior to the introduction of the electronic medical record system, Bru-HIMS. This may result in an underestimation and/or overestimation of CRC patients’ survival rates. In addition, CRC patients who chose to seek diagnosis and/or treatment overseas may not be enrolled in the registry (although this number is likely to be small due to the availability of free universal healthcare services in Brunei). In addition, in-situ cases were not included in the analysis as the data obtained from the cancer registry using CanReg5 was designed primarily for reporting of malignant cancers. The presence of unknown variables in the cancer registry may also lead to ambiguity in the results. Information such as patients’ family health history, comorbidities, diet, lifestyle factors, treatment(s) received and their responses to treatments were not available, thus were not evaluated. A larger data set including those from pre-2002 would enable an improved assessment of the survival rates of CRC patients in Brunei Darussalam (BDCR was only established in 2002, therefore prior data were not analysed).

In conclusion, this study evaluated the 1-, 3- and 5-year survival rates of CRC patients in Brunei, compared the survival rates between age groups, ethnicities, cancer stages, and sites of cancer, as well as compared CRC patients’ survival rates between two different study periods. Although the overall survival rates were similar to data from other countries/population, our study observed a significant increase in the number of new CRC cases in the period 2010–2017 compared to 2002-2009, increase in the proportion of younger CRC patients and a high proportion of CRC diagnosed at advanced stages. These findings highlight the importance of public health policies and programmes to enhance cancer prevention strategies and improve awareness on CRC including screening, symptom recognition among the younger population, healthy lifestyle practices, and the significance of early diagnosis and early intervention, to reduce CRC-associated mortality.

## Data Availability

The datasets used and/or analysed during the current study are available from the corresponding author on reasonable request.

## Abbreviations

Adj. HR(s): Adjusted hazard ratio(s)
ASR: Age-standardised incidence rate
Bru-HIMS: Brunei Darussalam Healthcare Information and Management System
CI(s): Confidence Interval(s)
CRC: Colorectal cancer
FIT: Faecal immunochemical testing
FIT-DNA: Faecal immunochemical testing-DNA test
FOBT: Faecal occult blood testing
HR: Hazard ratio
HDI: Human development index
IBD: Inflammatory bowel disease
LCRC: Left-sided colorectal cancer
MSI: Microsatellite instability
MT-sDNA: Multi-targeted stool DNA test
NOS: Not otherwise specified
RCRC: Right-sided colorectal cancer
SEER: Surveillance, epidemiology, and end results
